# Comparative study of AQP4-NMOSD, MOGAD and seronegative NMOSD: a single-center Belgian cohort

**DOI:** 10.1007/s13760-021-01712-3

**Published:** 2021-06-07

**Authors:** Solène Dauby, Dominique Dive, Laurence Lutteri, Cécile Andris, Isabelle Hansen, Pierre Maquet, Emilie Lommers

**Affiliations:** 1 Clinical Neuroimmunology Unit, Department of Neurology, CHU Liège, University Hospital of Liège, Liège, Belgium; 2 GIGA–CRC in Vivo Imaging, University of Liège, Liège, Belgium; 3 Clinical Ophthalmological Unit, Ophthalmology Department, University Hospital of Liège, Liège, Belgium; 4 Clinical Chemistry Department, University Hospital of Liège, Liège, Belgium

**Keywords:** Neuromyelitis optica, MOG-associated disorders, AQP4-antibody NMO spectrum disorders, Optic neuritis

## Abstract

**Purpose:**

To emphasize physio-pathological, clinical and prognosis differences between conditions causing serious and sometimes very similar clinical manifestations: anti-aquaporin-4 (AQP4) and anti-myelin oligodendrocyte glycoprotein (MOG) antibodies related diseases, and seronegative NMOSD (neuromyelitis optica spectrum disorders).

**Methods:**

Based on Wingerchuk et al. (Neurology 85:177–189, 2015) criteria for NMOSD and on those more recently proposed by Jarius et al. (J Neuroinflammation 15:134, 2018) for MOGAD (MOG associated disorders), we retrospectively surveyed 10 AQP4-NMOSD, 8 MOGAD and 2 seronegative NMOSD, followed at the specialized neuroimmunology unit of the CHU Liège.

**Results:**

Female predominance was only observed in AQP4 group. Age at onset was 37.8 and 27.7 years old for AQP4-NMOSD and MOGAD respectively. In both groups, the first clinical event most often consisted of optic neuritis (ON), followed by isolated myelitis. Fifteen of our 20 patients encountered a relapsing course with 90% relapses in AQP4-NMOSD, 62.5% in MOGAD and 50% in seronegative group, and a mean period between first and second clinical event of 7.1 and 4.8 months for AQP4-NMOSD and MOGAD, respectively. In total we counted 54 ON, with more ON per patient in MOGAD. MOG-associated ON mainly affected the anterior part of the optic nerve with a papilledema in 79.2% of cases. Despite a fairly good visual outcome after MOG-associated ON, retinal nerve fibre layer (RNFL) thickness decreased, suggesting a fragility of the optic nerve toward further attacks.

**Conclusion:**

As observed in larger cohorts, our MOGAD and AQP4-NMOSD cases differ by clinical and prognostic features. A better understanding of these diseases should encourage prompt biological screening and hasten proper diagnosis and treatment.

**Supplementary Information:**

The online version contains supplementary material available at 10.1007/s13760-021-01712-3.

## Introduction

In 1894, Eugène Devic reported atypical cases of multiple sclerosis (MS) characterized by an inflammatory lesion of both optic nerves and spinal cord, a condition which he dubbed ‘neuromyelitis optica’ (NMO) [1, 2]. NMO was considered as a subtype of MS until the discovery of causal antibodies against AQP4 protein [3, 4]. This objective biomarker allowed for a broadening of NMO phenotype to isolated myelitis without optic neuritis (ON) or even central nervous system (CNS) involvement without a spinal cord or optic nerve damage, e.g., *area postrema syndrome* [5]. This pleomorphic phenotype led to the concept of ‘NMO spectrum disorders’ (NMOSD) [6]. It later appeared that only 80% patients with a NMOSD phenotype are seropositive for AQP4-antibodies: other cases are seronegative and a minority of them (20%) bear antibodies against MOG [7–10]. Initially, MOG-antibodies were largely associated to several inflammatory diseases including MS, but the former western-blots and ELISA lacked specificity. Antibodies against conformational MOG epitopes detected by cell-based assay (CBA) were later causally implicated in acute disseminated encephalomyelitis (ADEM) and NMOSD.

The clinical phenotype of disorders with MOG-antibodies far exceeds neuromyelitis optica and includes uni- or bilateral, isolated or recurrent ON, myelitis with or without ON, ADEM [11], brainstem and supra-tentorial lesions [12–14]. These disorders are now referred to as ‘MOG-associated disorders’ (MOGAD). Finally, patients with NMOSD, negative for both AQP4 and MOG antibodies, are now diagnosed as ‘seronegative NMOSD’ (SN-NMOSD).

The distinction between MS, AQP4-NMOSD, MOGAD and SN-NMOSD is essential but challenging due to overlapping clinical, biological and radiological features [8]. A reliable diagnosis is mandatory to ensure proper treatment and prognosis. Here, we detail the clinical, biological and imaging phenotype of AQP4-NMOSD, MOGAD and SN-NMOSD patients diagnosed in the neuroimmunology unit of CHU Liège between 1978 and 2020.

## Methods

This monocentric retrospective study is based on the analysis of medical records of twenty patients from a local database. The cohort consists of 10 AQP4-NMOSD, 8 MOGAD and 2 SN-NMOSD. Before March 2011, AQP4-antibody detection was based on indirect immunofluorescence techniques on monkey cerebellum slices. From then on, Euroimmun CBA was used to test AQP4 locally, and Oxford CBA to test MOG in the Multiple Sclerosis and Neuromyelitis Group of the Oxford University [15].

Summary statistical analysis consisted of medians and percentiles. Non-parametric tests (Kruskal–Wallis and *χ*^2^ tests) were used for group comparisons.

## Results

### Epidemiological findings

There was a female dominance in AQP4-NMOSD (F/M = 10/0; *χ*^2^(1) = 20, *p* < 0.0001), which was not found in MOGAD (F/M = 4/4). Age at first clinical event significantly varied across groups (Kruskal–Wallis, *χ*^2^(2) = 6.77, *p* = 0.033), showing earlier onset in MOGAD patients than in AQP4-NMOSD (*χ*^2^(1) = 4.18, *p* = 0.041). Median age at the first clinical event was 37.8 years in AQP4-NMOSD (range 14–73.7) and 27.7 years in MOGAD (range 9.8–39.5). The two seronegative patients had their first clinical event, respectively, at 63 and 45 years.

### Initial clinical event

The initial clinical event in AQP4-NMOSD was unilateral ON (*N* = 4), myelitis (*N* = 3), bilateral ON (*N* = 2), or simultaneous myelitis and ON (*N* = 1). In MOGAD, the initial clinical event in adults was either a bilateral (*N* = 4) or unilateral (*N* = 3) ON, or an isolated myelitis (*N* = 1). In all cases, inaugural myelitides were longitudinally extensive (≥ 3 vertebral segments) transverse myelitides (LETM) [5]. AQP4-NMOSD myelitides were located in the lower cervical cord and usually expanded to thoracic levels (down to T9). The MOGAD LETM affected the upper cervical cord with extension to the lower medulla.

### Misdiagnosis and delayed diagnosis

The diagnosis of AQP4-NMOSD or MOGAD is demanding due to the wide differential diagnosis involving other CNS inflammatory conditions. In our 20 patients, the median time between the initial clinical event and diagnosis was 54 months (4.5 years). It is interesting to note that after 2012 (and the advent of CBA for antibodies testing), median time for diagnosis was substantially shorter (7.4 months, *χ*^2^(1) = 12.18, *p* = 0.0005; supplemental figure 1). Delayed diagnosis did not depend on final diagnosis (*χ*^2^(2) = 2.45, *p* = 0.29).

Erroneous initial diagnoses included MS (*N* = 3), isolated or relapsing inflammatory optic neuritis (CRION) (*N* = 5), pseudotumor cerebri (*N* = 2), ischemic optic neuropathy (*N* = 1), lupus-related myelitis (*N* = 1), syringomyelia (*N* = 1), suspected lymphomatous infiltrate (*N* = 1), iatrogenic myelitis after anti-TNF alpha treatment (*N* = 1) [16], neurotuberculosis (*N* = 1) and Harding syndrome (combination of MS and Leber hereditary optic neuropathy; *N* = 1). None of the MOGAD patients fulfilled the 2017 McDonald MS diagnostic criteria [17] whereas 8/10 AQP4-NMOSD patients did.

### Biological findings

CSF analysis showed lymphocytic pleocytosis in 4/9 AQP4-NMOSD and in 2/8 MOGAD (*χ*^2^(1) = 0.70, *p* = 0.40) as well as CSF oligoclonal bands in 3/10 AQP4-NMOSD (persisting in 2 patients at later CSF control) and no MOGAD (*χ*^2^(1) = 2.88, *p* = 0.09).

Half (5/10) of AQP4-NMOSD patients and 2/8 MOGAD patients showed biological signs of auto-immunity (*χ*^2^(1) = 1.17, *p* = 0.28). Antinuclear antibodies were found in all five AQP4-NMOSD patients (4 patients, 1/320; one patient 1/640; 1 anti-SSA/Ro, 1 anti-ribosome, 1 anti-centromere, 2 without characterization). One of them had additional autoantibodies (anti-myeloperoxydase antineutrophil cytoplasm antibodies and anti-gastric mucosa). The anti-SSA-positive patient was diagnosed with systemic lupus erythematosus (SLE). MOGAD patients had either antinuclear uncharacterized antibodies (1/160) and psoriasis (1 patient) or anti-GM1 IgM antibodies, without any clinical manifestation (1 patient).

### Disease course: monophasic versus relapsing?

The median length of follow-up was 7.5 years with a minimum of 1.4 and a maximum of 32.9 years. Fifteen patients suffered at least one relapse (supplemental figure 2). Relapses were more prevalent in AQP4-NMOSD than in MOGAD: we observed relapses in 9/10 AQP4-NMOSD, 5/8 MOGAD and 1/2 seronegative patients. However, the difference in relapse rates between groups was not significant (*χ*^2^(1) = 0.81, *p* = 0.67).

**Fig. 1:**
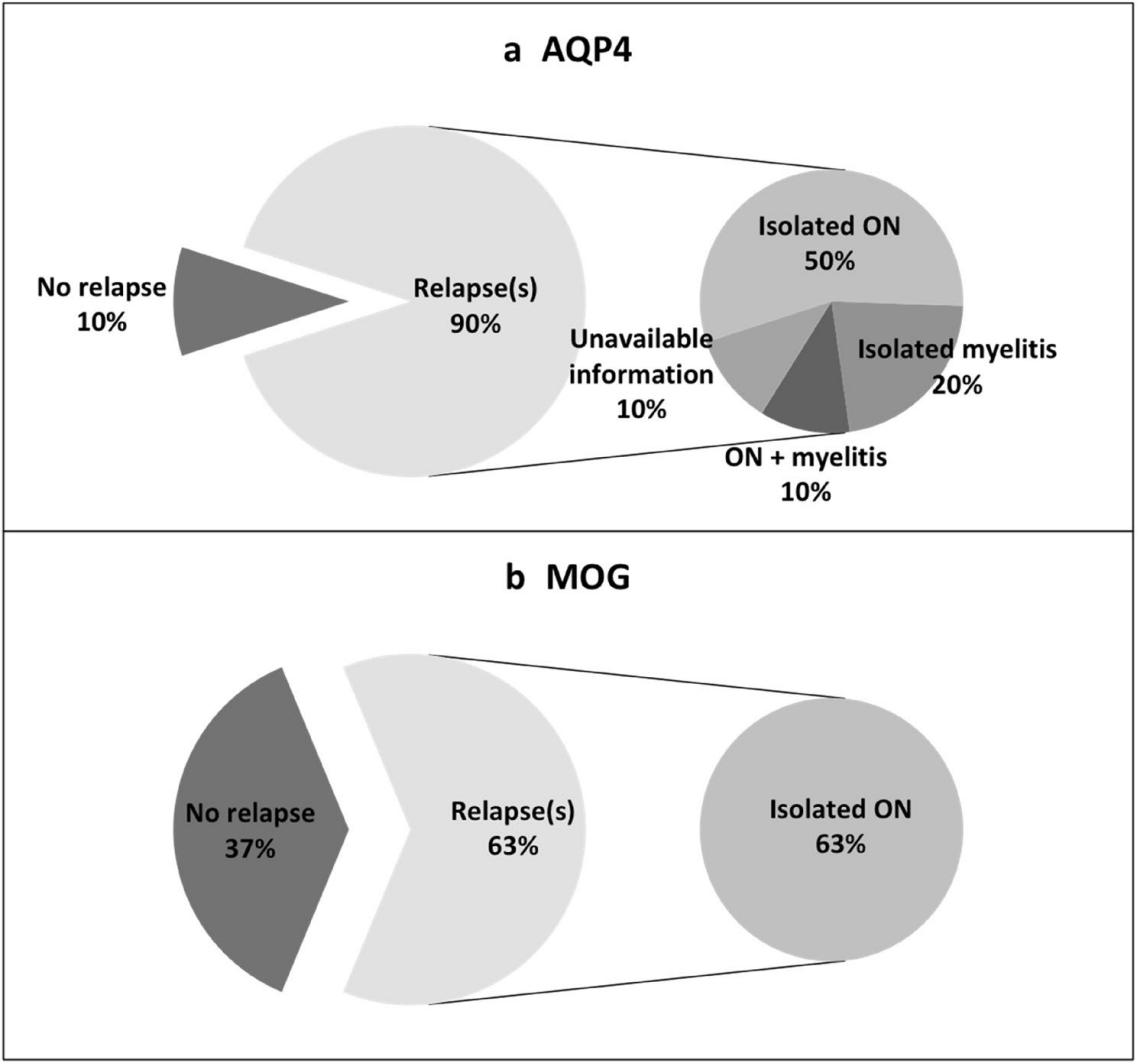
Clinical relapses in AQP4 NMOSD group (**a**) and MOGAD group (**b**). Right-hand side charts represent the proportion of different clinical manifestation

First relapse consisted of ON for all MOGAD (5/5) and for 5/9 AQP4-NMOSD patients. Two AQP4-NMOSD patients relapsed with isolated myelitis and one relapsed with simultaneous unilateral ON and myelitis. Relapse description is unavailable for the last NMOSD-AQP4 patient (Fig. 1). The mean interval between the first and second clinical event did not differ between groups: 7.1 months for AQP4-NMOSD and 4.8 months for MOGAD (*χ*^2^(1) = 0.77, *p* = 0.38).

In AQP4-NMOSD group, several relapses occurred despite a first-line immunosuppressive treatment in 6/9, leading to therapeutic escalation. By contrast, 2/8 MOGAD patients relapsed after treatment initiation. The MOG patient M1 relapsed 1.5 months after initiation of mycophenolate mofetil (MMF, 1000 mg bid) with a second ON. He was then treated by rituximab, which did not prevent 3 additional ON. Monthly perfusions of off-label intravenous immunoglobulins (IVIg) stopped relapses for 12 months, so far. Patient M6 received MMF (1000 mg bid) after four episodes of ON and suffered an additional ON 6 months after treatment initiation, probably due to suboptimal treatment adherence.

### Seronegative patients

Two female patients fulfilled the Wingerchuk 2015 diagnostic criteria for SN-NMOSD [5] (N1, N2, supplemental figure 2). AQP4 and MOG antibodies were both tested several times, including during clinical relapses, with no avail.

**Fig. 2:**
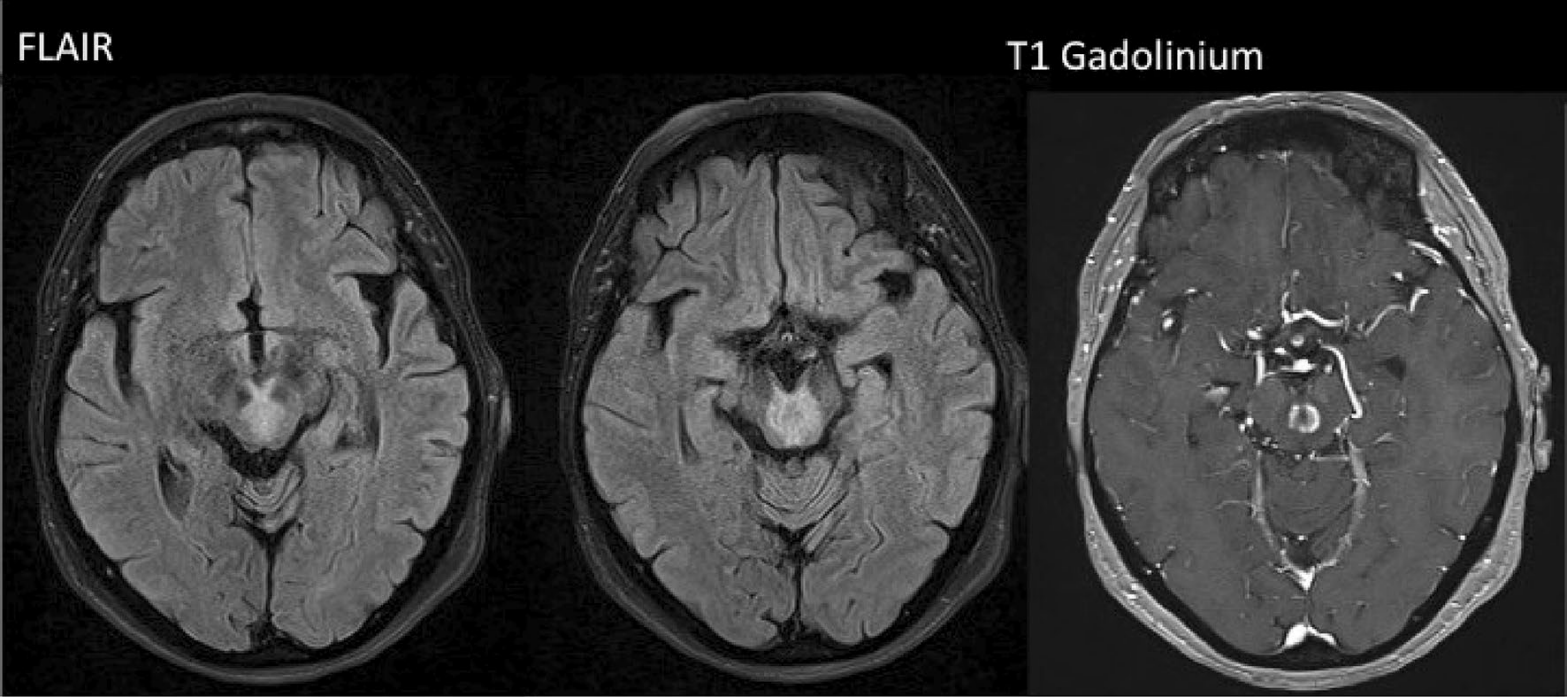
Patient 1 (N1) brain MRI. Note the central midbrain lesion with contrast enhancement

**Fig. 3:**
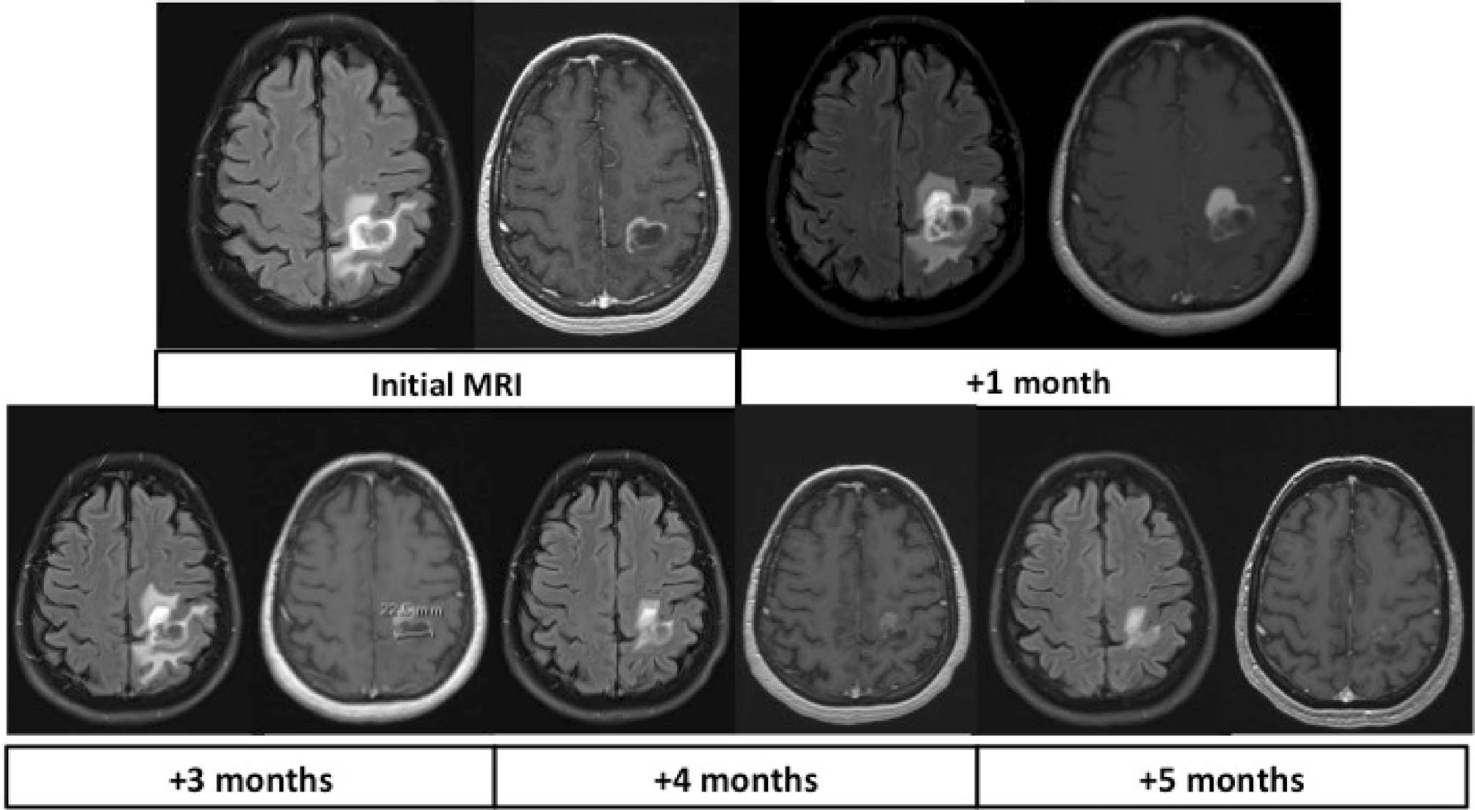
Patient 1 (N1) brain MRI (FLAIR on the left and T1 gadolinium on the right) showing progressive reduction of pseudotumoral lesion size and contrast enhancement over time, while receiving rituximab infusions

The first patient (N1) had two severe ON, 1 year apart, affecting both eyes with complete recovery after intravenous (IV) corticoids. Four years later, she developed dysarthria, diplopia and left facial palsy attributable to a ponto-mesencephalic inflammatory lesion (Fig. 2). Seven plasma exchanges (PLEX) followed by a long-term treatment with MMF (1000 mg bid) resulted in an excellent recovery. A third relapse, a right hemiparesis, occurred 1 year later, due to a left-hemispheric pseudotumoral lesion (Fig. 3). Transient improvement was observed after IV corticoids and a first rituximab cycle but a new clinical impairment associated with lesion growth occurred after 8 weeks. Ten PLEX and the continuation of rituximab (one cycle every 6 months) resulted in stable clinical and radiological improvement. After a 2-year follow-up, the patient is pauci-symptomatic and no relapse occurred.

The younger patient (N2) suffered a severe bilateral asymmetric ON (visual acuity at 1 and 5/10) with cervical myelitis at the age of 45 years. She received 1 g IV corticoids for 5 days, followed by six PLEX, two-month treatment by azathioprine, then a single cycle of rituximab (1 g 2 weeks apart) and a long-term treatment by MMF (1000 mg bid). She is currently relapse-free for 3.5 years.

### Optic neuritis

Eighteen of our 20 patients had at least one ON. For 16 of them, it was the first, and in about half of the cases, the sole clinical event. In total, we documented 54 ON (Table 1). If we consider the number of ON relapses by a patient, we observe more relapses per patient in the MOG group (4.25 relapses) than in AQP4 group (3 relapses). There was not any significant difference in relapse rate between groups, as 6 AQP4 patients out of 8 had several optic neuritis, for only 4 MOG patients out of 8 (*χ*^2^(2) = 0.61, *p* = 0.74). The rate of bilateral ON was not different between groups (4 bilateral ON in both groups for 21 and 22 unilateral ON in AQP4 and MOG groups, respectively; *χ*^2^(2) = 0.0263, *p* = 0.99).

**Table 1:** Optic neuritis (ON) and relapses in each group

	AQP4	MOG	Seronegative	Total
Patients with ≥ 1 ON	8/10 (80%)	8/8 (100%)	2/2 (100%)	18/20 (90%)
Total number of ON	26	25	3	54
Unilateral ON	22 (84.6%)	21 (84%)	2 (66.7%)	45
Bilateral ON	4 (15.4%)	4 (16%)	1 (33.3%)	9
Patients with ≥ 1 relapse of ON	6/8 (75%)	4/8 (50%)	1/2 (50%)	11/18 (61.1%)
Average number of relapse if relapse(s)	3	4.25	1	*–*

**Fig. 4:**
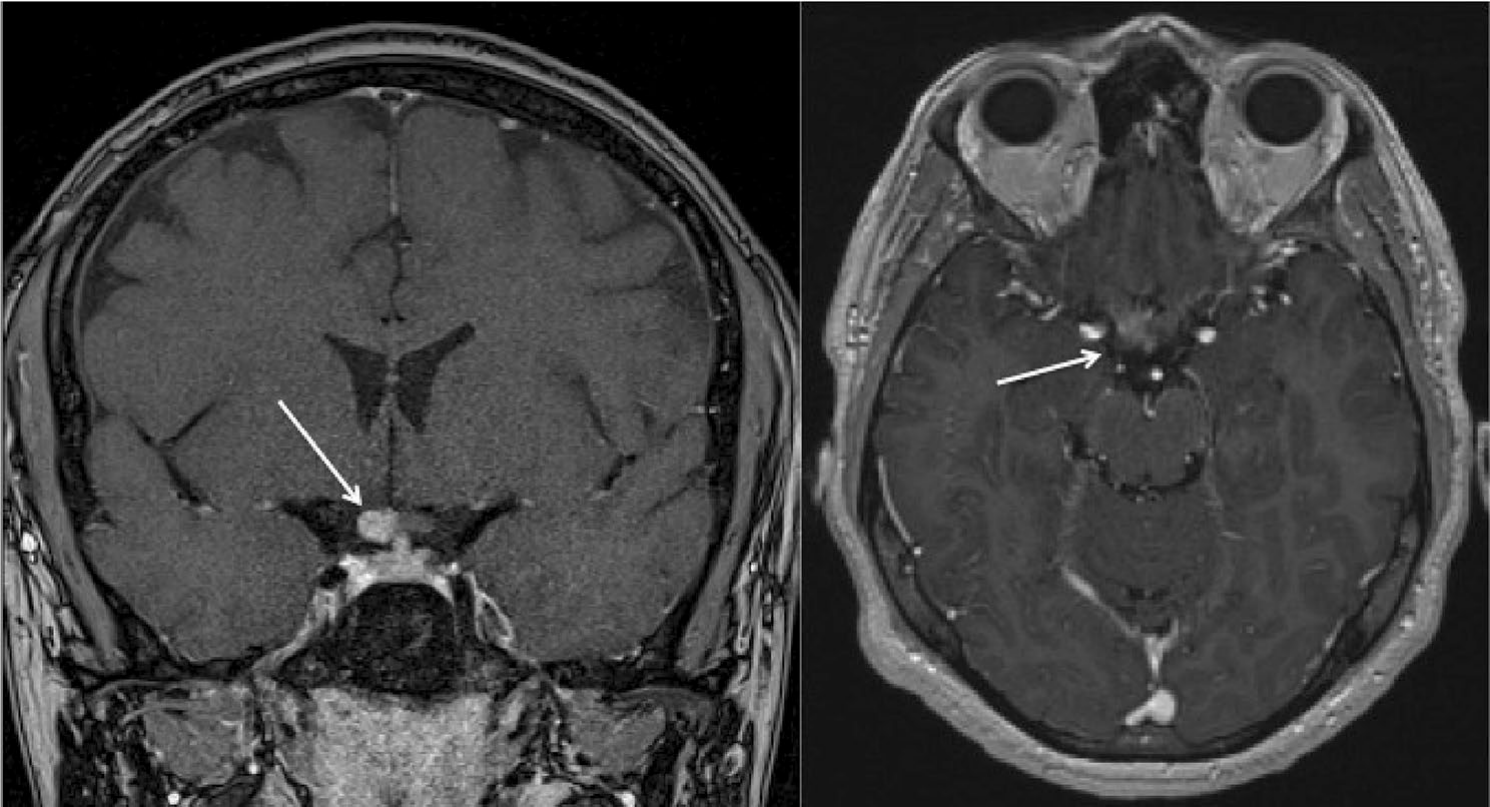
Brain MRI showing a lesion of the optic chiasm in a MOGAD patient (M2), with contrast enhancement

The anterior part of the optic nerve was preferentially injured in anti-MOG neuritis with 19/25 ON associated with papilledema on the fundus examination. By contrast, one MOG patient encountered atypical attack of the optic chiasm (M2, Fig. 4).

The visual outcome of AQP4-NMOSD encountering ON was variable and based on fragmentary information: 3/8 had poor (< 1/10) bilateral visual acuity (VA), 2/8 poor unilateral VA, 2/8 moderate (< 8/10) unilateral VA and 1/8 good visual outcome.

**Fig. 5:**
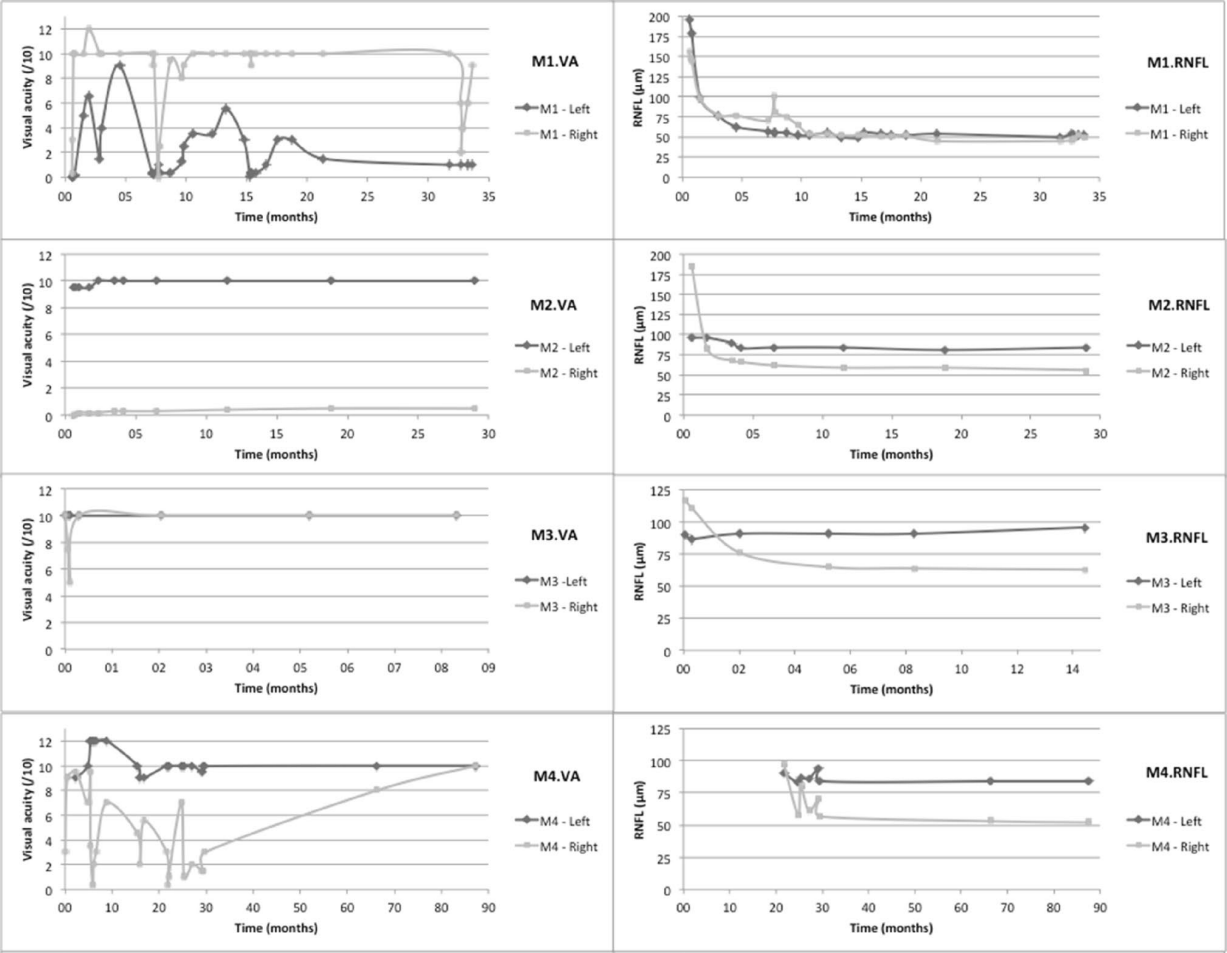

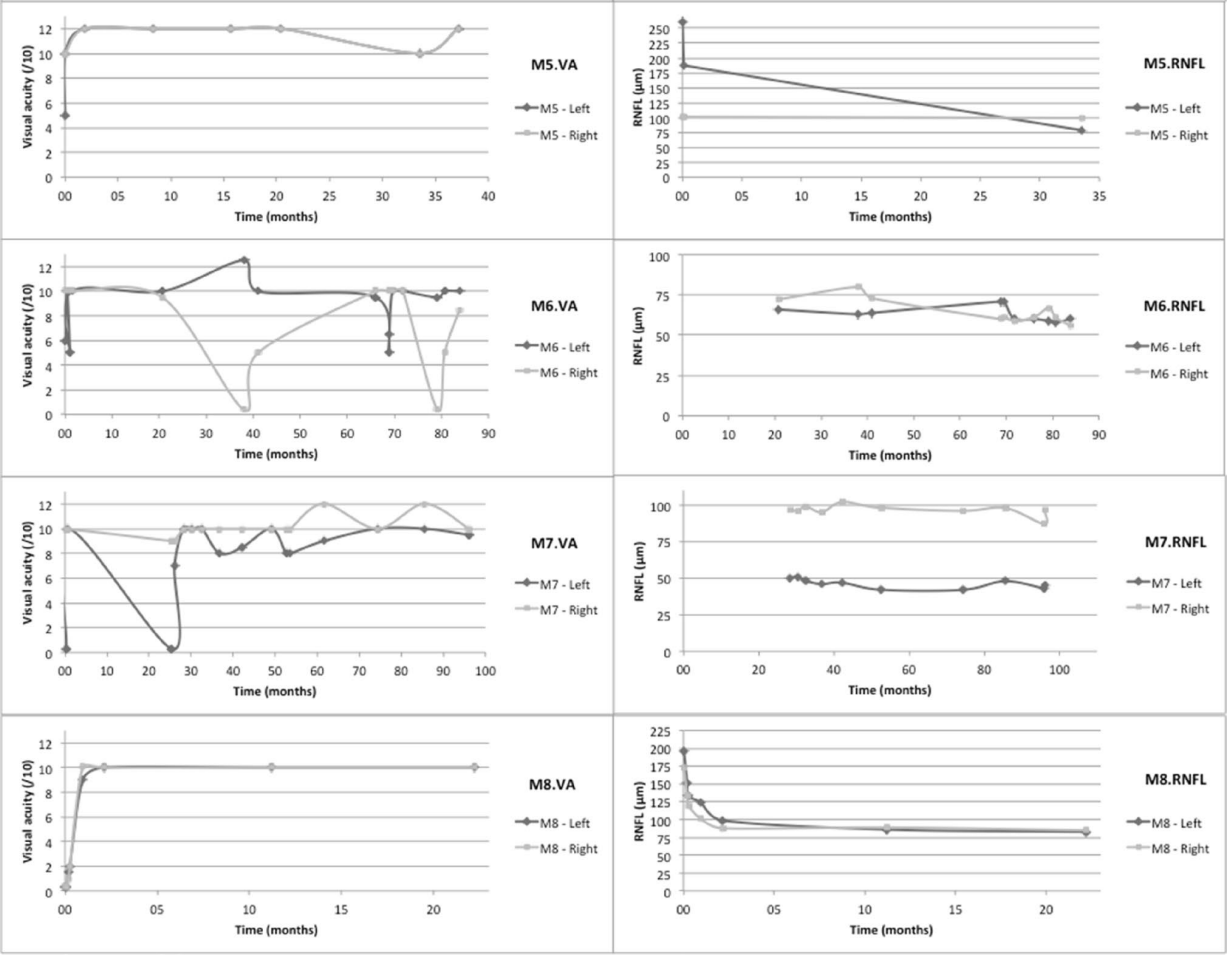
Charts on the left-hand side represent visual acuity of both eyes over time for each MOGAD patients and charts on the right-hand side represent RNFL thickness measured by OCT over time. RNFL thickness is clearly reduced for optic nerves that were injured by inflammation, and this slimming is obvious even when visual acuity shows a good recovery. This is particularly the case for patients M3, M4 and M7 whose visual acuities are bilaterally assessed at 10/10 by the end of follow-up but whose optic nerve thickness are distinctly altered

VA and RNFL thickness courses have been more systematically assessed in the 8 MOGAD patients (Fig. 5). VA was most of the time well preserved, in contrast to our AQP4-NMOSD patients. Indeed, all MOGAD patients had VA higher or equal to 8.5/10, even after repeated and/or severe attacks, except two patients who kept a unilateral VA below or equal to 1/10. Figure 5 illustrates the VA and RNFL thickness of MOGAD patients over time while they received corticoids or PLEX during the acute phase and maintenance immunotherapies (azathioprine, MMF, rituximab or IVIg). Four out of 8 MOG-patients (Table 1) encountered only one ON and did not relapse under immunotherapy: M2, M3, M5 and M8 of Fig. 5.

Among the 4 MOG-patients who experienced at least one ON relapse: M1 patient relapsed under MMF, leading to therapeutic escalation by rituximab and then by IVIg. M4 patient only received azathioprine after 6 ON event but it did not prevent the seventh one, leading to methotrexate and then rituximab introduction. As mentioned above, M6 patient relapsed under MMF treatment because of a suboptimal treatment adherence. Finally, M7 patient received MMF after 3 ON and no relapse has happened since then.

## Discussion

Neurological syndromes associated with MOG- and AQP4-antibodies now appear as two separate demyelinating diseases, characterized by distinct pathophysiology: AQP4-NMOSD is an astrocytopathy while MOGAD currently appears as an oligodendrogliopathy [18]. Although they may share similar clinical phenotype, epidemiological and clinical specificities were previously described in larger cohorts.

In keeping with the literature [8, 11, 18–26], gender and age at onset were significantly different between groups with a clear female prevalence in AQP4-NMOSD and a younger age of onset in MOGAD. Co-existing auto-antibodies were observed in both groups, but more frequently with AQP4-NMOSD than with MOGAD as reported in the literature [27–30]. Seropositivity for autoantibodies, mainly antinuclear, can reach 38 to 75% in NMOSD patients whether AQP4 positive or seronegative [28]. SLE and Sjögren syndrome are the most prevalent associated autoimmune diseases in AQP4-NMOSD, followed by myasthenia gravis and thyroiditis. The reasons explaining the differential proportion of autoimmune comorbidities between AQP4-NMOSD and MOGAD remain unclear: whereas AQP4-NMOSD results from abnormal B-cell tolerance checkpoint, MOGAD would rather be due to molecular mimicry [30].

The initial clinical event in AQP4-NMOSD is, by decreasing frequency, unilateral ON, myelitis, bilateral ON, myelitis plus ON, brainstem (mainly an *area postrema syndrome*) and supra-tentorial lesions [5, 31]. In MOGAD, the first clinical event depends on the age of onset [22]. ADEM and encephalopathy predominate in the paediatric population while adults are more likely to suffer from myelitis or ON. Brainstem, cerebral parenchyma or cerebellar attacks as well as multifocal lesions have also been described [22, 23, 32]. ON was the most represented first clinical event in our entire cohort (more often bilateral in anti-MOG than anti-AQP4 in larger cohorts than ours [12, 33]), followed by myelitis and the simultaneous attack of both regions. This is consistent with earlier studies [24, 26, 33–35]. We noted the conspicuous absence of ADEM in our cohort, which only included two children under the age of 16. Moreover, adult forms of ADEM are less frequently associated with anti-MOG seropositivity [36].

ON was the most common manifestation for both conditions. Compared to typical MS and idiopathic ON, optic nerve attacks in AQP4-NMOSD and MOGAD are more severe, more often bilateral, extensive and associated with perineural enhancement [11, 37, 38]. Antibodies testing must therefore be considered in all cases of atypical ON. Beside biological clues, the lesion topography, as assessed by ophthalmological examination and magnetic resonance imaging, also helps distinguishing AQP4-NMOSD from MOGAD associated ON. Anterior part of the optic nerve is preferentially injured in anti-MOG neuritis, whereas in AQP4-neuritis, it rather concerns the posterior part of the visual pathways [37, 39]. Fundus examination sometimes shows papillitis or papilledema suggestive of an anterior neuritis. However, this finding might not be sensitive enough for the detection of a slight papilledema, for which an optical coherence tomography (OCT) is needed. Papilledema was observed in 85% of MOG-associated ON described in the literature [40] and in 79.2% of our cases (19/25). This finding can be misleading and suggest pseudotumor cerebri—for bilateral attack—or an anterior ischemic optic neuropathy—for unilateral attack, as it was the case for some of our patients. It however should be kept in mind that posterior and even chiasmatic attack may occur in 5–15% of MOG-associated ON [23, 37, 38, 41, 42], as in patient M2 (Fig. 4).

Existing literature indicates that ON relapse more frequently in MOGAD than in AQP4-NMOSD [11, 35]. This trend was not observed in our cohort, as 75% of our AQP4 patients (6/8) have undergone several ON, for only 50% in the MOG group (4/8). However, within patients who relapsed, the number of ON relapses per patient was higher in the MOG group (4.25 relapses) than in the AQP4 group (3 relapses). The rate of bilateral attacks was not significantly different between groups.

Functional prognosis remains good in the MOG group: follow-up typically showed a good VA recovery after anti-MOG neuritis, in keeping with previous studies [11, 24, 25, 33, 39, 40, 43, 44]. Nevertheless, the progressive and irreversible atrophy of the optic nerve, as measured by the RNFL thickness with OCT, is a compelling argument to treat patients as early as possible. Concordant with the literature, our sample of AQP4-NMOSD patients showed a worse visual outcome. However, delayed diagnosis and treatment as compared to MOG patients (supplemental figure 2) can in part explain this result.

AQP4-NMOSD and MOGAD usually follow a relapsing course but, in contrast to MS, do not show any significant progression [8, 45]. Most of our patients (15/20) suffered clinical relapses, with no significant difference in relapse rates or interval between the first and second event between our two groups. By contrast, the literature indicates a relapse rate lower in MOGAD than in AQP4-NMOSD, and related to MOG antibodies titre [23, 24]. Antibody levels increase during disease activity but decrease (and can become undetectable) between relapses as well as under immunosuppressive treatment [19, 23, 24]. Based on this observation, treatment was discontinued in two patients (M3, M5) after they turned out to be seronegative for MOG-antibody. They did not relapse so far for two and one year and a half, respectively. As suggested in Ref. [46], antibody titres are monitored every 6 or 12 months and are still undetectable.

Two patients in our cohort were seronegative for both AQP4 and MOG antibodies and fulfilled diagnostic criteria for SN-NMOSD [5]. They encountered a favourable outcome with good clinical recovery after prompt and appropriate treatment. While SN-NMOSD are more likely monophasic diseases and associated with less severe clinical attack [31], N1 seronegative patient experienced 3 severe relapses, including a vast pseudotumoral lesion of the left sensorimotor cortex (Fig. 3).

Before the advent of CBA for antibodies testing in 2012, the diagnosis of NMOSD or MOGAD was challenging due to a large number of alternative diagnoses. In our entire cohort, the median time between initial clinical event and diagnosis was 54 months (4.5 years), with a substantial reduction of this period (7.4 months) when considering patients whose first clinical event occurred after 2012. This finding emphasizes the diagnostic advance provided by CBA testing.

From our experience, earlier diagnosis led to prompt treatment and better clinical outcome. Because MOGAD and AQP4-NMOSD are relapsing conditions, a preventive immunosuppressive treatment must always be considered at the time of diagnosis. Moreover, the importance of an accurate differential diagnosis between MOGAD and AQP4-NMOSD is becoming crucial. While acute management consists of intravenous high doses of corticosteroids and PLEX (especially for refractory conditions) for both diseases, chronic treatment should differ. Monoclonal anti-CD20 rituximab seems considerably more efficient in AQP4-NMOSD than in MOGAD, with 80–90% response instead of ~ 30%, respectively [47, 48]. Eculizumab, inebilizumab and satralizumab in AQP4-NMOSD seem associated with good outcomes [49, 50]. Interleukin 6 pathway inhibitors or complement blocking therapies have been tried in some MOGAD patients [51] but dedicated clinical trials are not available yet. For this condition, no standardized therapy is recommended. Recent publications suggest that IVIg should be considered in MOGAD while a positive effect was observed in one paediatric series [52, 53]. Four of our MOGAD patients were only treated by MMF (M3, 5, 6, 7), two received directly rituximab (M2, 8) and one received rituximab after azathioprine and methotrexate (M4). The last one was first treated by MMF but required a therapeutic escalation with rituximab then IVIg (M1).

## Conclusion

This retrospective analysis confirms that AQP4-NMOSD and MOGAD do not only differ by aetiology, but also by some clinical features and prognosis. As compared to MOGAD, AQP4-NMOSD is typically more frequent in females, occurs at an older age, involves the posterior optic nerve and relapses more frequently.

Practitioners should not hesitate to resort to ophthalmological assessment and to relevant biological screening in suggestive cases for promptly orienting the diagnosis, thereby avoiding severe disability observed during the natural course of these diseases.

## Supplementary Information

Below is the link to the electronic supplementary material.

Supplementary file1 Supplemental figure 1. Delay period before formal diagnosis depending on year of onset. In a chart, note the isolated component corresponding with the case that first clinical event occurred in 1978 and have the longer time taken to diagnosis. Note in b chart a significant delay period reduction from 2012 to the present day. (PNG 54 kb)

Supplementary file2 Supplemental figure 2. Left-hand side panel: timeline of clinical events and chronic treatments for each patient. Right-hand side panel: last clinical status at the end of follow-up. End of follow-up corresponds to death for A6, A7 and A9, lost sight for M8 and to December 2020 for the others. Note that A6 patient’s clinical events from 1978 to 2000 are not represented but consisted in 3 ON and 3 MY. A3 patient suffers from SLE and has been treated with plaquenil from 2006 to 2020, except during a one-year clinical trial in 2008 for which belimumab was used instead. 0: blindness, Ax : NMOSD-AQP4, CF: counting fingers, EDSS: Expanded Disability Status Scale, LP: light perception, Mx : MOGAD, MY: myelitis, MY+ON: simultaneous myelitis and optic neuritis, Nx : SN-NMOSD, O: other lesion topography (thalamic lesion for A3, parenchymal lesion for A6, ponto-mesencephalic and parenchymal lesions for N1), ON: optic neuritis, VA: visual acuity/10 (left-right). (PNG 62 kb)

## Data Availability

The data that support this study are available on request from the corresponding author. The data are not publicly available due to privacy or ethical restrictions.
