# Supplementary figures and images for: Comparative study of AQP4-NMOSD, MOGAD and seronegative NMOSD: a single-center Belgian cohort
Source: Acta Neurol Belg. 2021 Jun 7;122(1):135–44. doi: 10.1007/s13760-021-01712-3 (PMC8894224; doi:10.1007/s13760-021-01712-3)

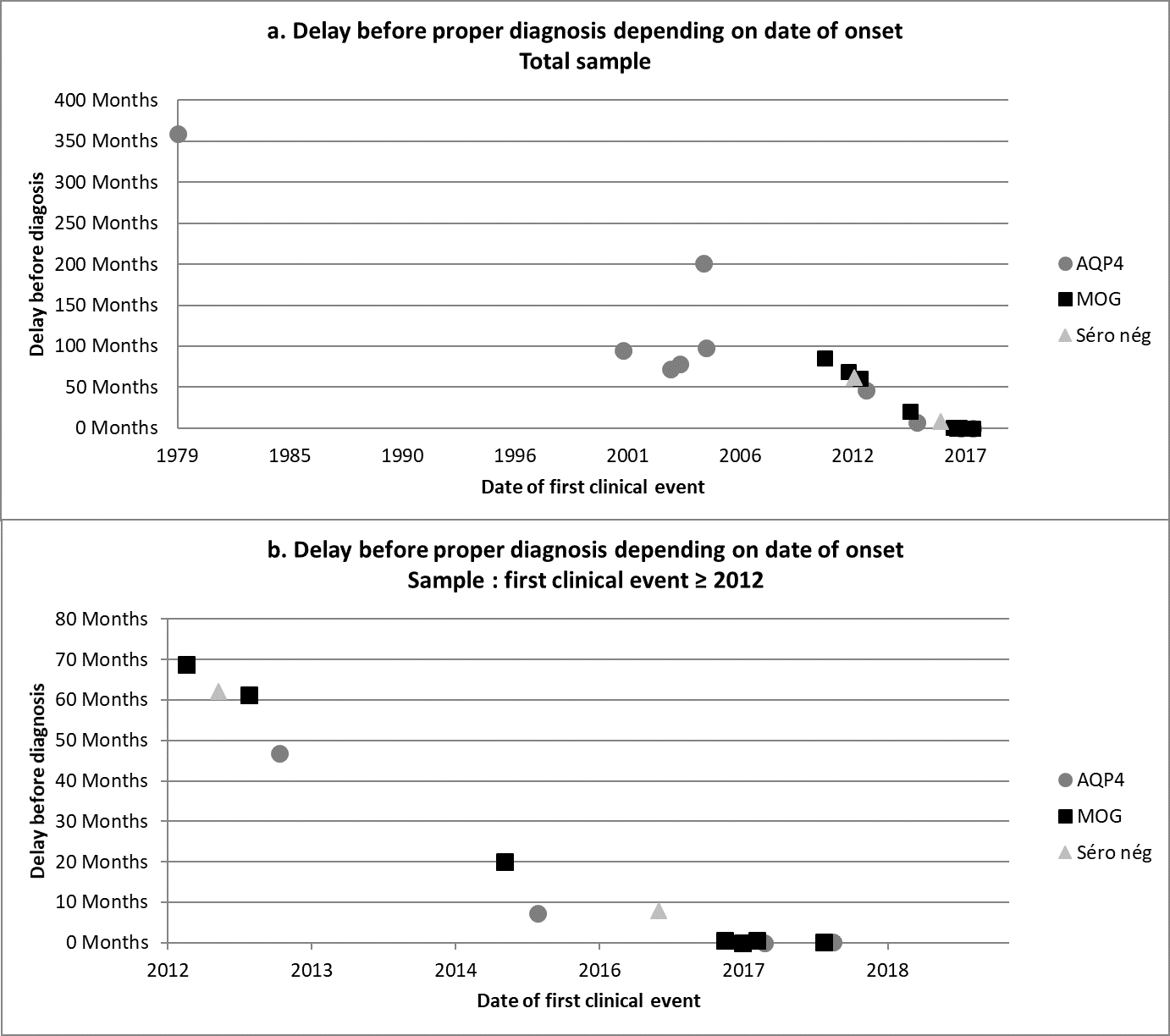

Supplement: Supplementary file 1 — Supplementary file1 Supplemental figure 1. Delay period before formal diagnosis depending on year of onset. In a chart, note the isolated component corresponding with the case that first clinical event occurred in 1978 and have the longer time taken to diagnosis. Note in b chart a significant delay period reduction from 2012 to the present day. (PNG 54 kb) [file 13760_2021_1712_MOESM1_ESM.png]

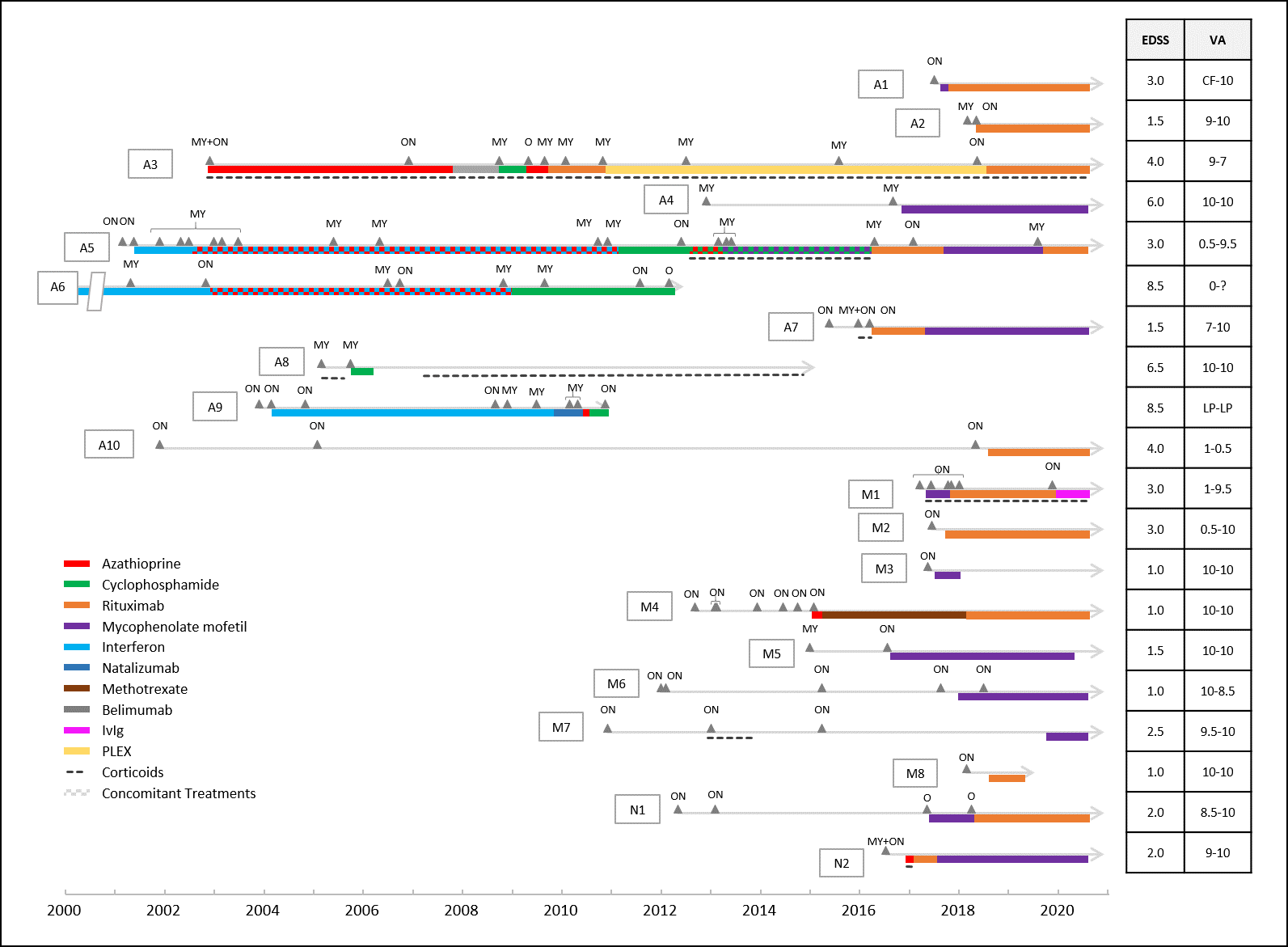

Supplement: Supplementary file 2 — Supplementary file2 Supplemental figure 2. Left-hand side panel: timeline of clinical events and chronic treatments for each patient. Right-hand side panel: last clinical status at the end of follow-up. End of follow-up corresponds to death for A6, A7 and A9, lost sight for M8 and to December 2020 for the others. Note that A6 patient’s clinical events from 1978 to 2000 are not represented but consisted in 3 ON and 3 MY. A3 patient suffers from SLE and has been treated with plaquenil from 2006 to 2020, except during a one-year clinical trial in 2008 for which belimumab was used instead. 0: blindness, Ax : NMOSD-AQP4, CF: counting fingers, EDSS: Expanded Disability Status Scale, LP: light perception, Mx : MOGAD, MY: myelitis, MY+ON: simultaneous myelitis and optic neuritis, Nx : SN-NMOSD, O: other lesion topography (thalamic lesion for A3, parenchymal lesion for A6, ponto-mesencephalic and parenchymal lesions for N1), ON: optic neuritis, VA: visual acuity/10 (left-right). (PNG 62 kb) [file 13760_2021_1712_MOESM2_ESM.png]
